# Induction with interleukin-2 antagonist for transplantation of kidneys from older deceased donors: an observational study

**DOI:** 10.1186/2047-1440-2-11

**Published:** 2013-06-26

**Authors:** Kristian Heldal, Solveig Thorarinsdottir, Anders Hartmann, Torbjørn Leivestad, Anna V Reisæter, Aksel Espen Foss, Karsten Midtvedt

**Affiliations:** 1 Clinic of Internal Medicine, Telemark Hospital, 3710, Skien, Norway; 2 Section of Nephrology, Oslo University Hospital Rikshospitalet, Oslo, Norway; 3 Faculty of Medicine, University of Oslo, Oslo, Norway; 4 Section of Transplant Surgery, Oslo University Hospital Rikshospitalet, Oslo, Norway

**Keywords:** Kidney transplantation, Expanded criteria donor, Induction treatment, Age, Survival, Rejection

## Abstract

**Background:**

The most important limiting factor in kidney transplantation is the scarcity of donor organs. Consequently, there is an increased use worldwide of kidneys from older deceased donors. High donor age is a known risk factor for acute cellular rejection and premature graft failure, and the optimal immunosuppressive regimen in these circumstances remains to be established.

**Methods:**

We investigated whether induction treatment with an interleukin 2 (IL-2) receptor antagonist improves graft survival and reduces rejection episodes in recipients of kidneys from deceased donors aged ≥ 60 years. Data were retrieved for all recipients transplanted at our center from 2004 to 2009 with a kidney from a deceased donor aged > 60 years. The outcome was compared between recipients treated with (IL-2 plus) or without (IL-2 minus) an IL-2 receptor antagonist. All recipients received a calcineurin inhibitor, steroids and mycophenolate.

**Results:**

A total of 232 first-transplant recipients were included (IL-2 plus = 149, IL-2 minus = 83). IL-2 minus was associated with increased risk of early acute rejection (OR 2.42; 95% CI 1.25 to 4.68, *P* = 0.009) and steroid-resistant rejection (OR 8.04; 2.77 to 23.25, *P*< 0.001). IL-2 plus patients had superior two-year estimated uncensored (87% versus 70%, *P* = 0.001) and death-censored (95% versus 79%, *P*< 0.001) graft survival.

**Conclusions:**

Induction treatment with IL-2 receptor antagonist was associated with a reduction in acute rejection episodes and improved two-year graft survival in patients transplanted with kidneys from older deceased donors.

## Background

Both the incidence and the age of patients with end stage renal disease (ESRD) starting renal replacement therapy have increased dramatically during the last 20 to 30 years [1-4]. In selected older patients, kidney transplantation is safe and leads to superior results compared with continuous dialysis [5].

As the waiting lists grow, there is an ongoing debate concerning the scarcity of organs and whether it is appropriate to use such organs in high-risk recipients, especially in recipients of advanced age. Increased utilization of expanded criteria donors (ECD) may be a way to enlarge the donor pool. An ECD is per definition a deceased donor who is older than 60 or is 50 to 59 with at least two of three medical criteria: hypertension, cerebrovascular cause of death or serum creatinine level higher than 132.6 μmol/L [6]. This definition is based on organs for which the characteristics were associated with a relative risk > 1.7 compared with normotensive donors aged 10 to 39 years for the outcome of overall transplant loss in a model generated by the Scientific Registry of Transplant Recipients and published in 2002 [6]. With recent developments in immunosuppression [7,8], recovery of organs and graft preservation [9], the overall outcomes for recipients of ECD kidneys may have improved and those variables found to give increased risk may have been modified. An important issue in the process of allocation of donor organs is to match the expected life span of the transplanted organ to the expected life span of the recipient. An old-for-old allocation strategy has been used as a tool for matching the organ with the recipient’s needs and thereby giving older ESRD patients a possibility of receiving a transplant without displacing younger patients from the waiting list [10].

The optimal immunosuppressive regimen for kidney transplantation in an old-to-old setting is not established [11]. Older recipients have a less active immune response and consequently develop fewer acute rejection episodes than younger recipients [12]. Accordingly, some authors have proposed that older recipients may need less intense immunosuppression, thus avoiding potentially detrimental side effects such as serious infections or malignancies [13]. However, kidneys from older deceased donors may be more immunogenic [14,15]. This is believed to be the result of non-specific injuries that induce a pro-inflammatory milieu which, in turn, may activate immune responses [16]. In the Eurotransplant Senior Program, only patients who were transplanted with kidneys from older donors (> 65 years) had a significantly increased rate of acute rejection episodes and only about half of these patients received induction treatment (IL-2 receptor antagonist or T-cell depletion) [10]. The optimal level and type of immunosuppression in recipients of kidneys from older donors need to be clarified [5,11].

The aim of our study was to investigate whether there was any change in outcome after the introduction of induction treatment with an IL-2 receptor antagonist in recipients of first kidney transplants of organs from deceased donors over 60 years of age.

## Methods

The study was approved by Oslo University Hospital and by the regional committee for medical and health research ethics. All patients included gave their consent for the use of their clinical data in research. Clinical and survival data for all patients who received a kidney transplant from a deceased donor older than 60 years at Oslo University Hospital between 2004 and the end of 2009 were retrieved from the Norwegian Renal Registry. The last update of the survival data was performed on 31 December 2012. Patients not treated with a calcineurin inhibitor (CNI) as part of the initial regimen were excluded. Outcome variables including serum creatinine values for each individual are reported yearly to the registry. Information about immunosuppression reflects the initial regimen used by each patient and that at discharge from the transplant centre after 10 weeks. Time on dialysis was defined as the time from start of chronic dialysis to the time of transplantation. Delayed graft function (DGF) was defined as the need for dialysis the first week after transplantation and included patients with primary non-function. All acute rejection episodes were registered, and the vast majority were biopsy proven. HLA mismatch was analyzed as no mismatch versus any mismatch for HLA-A, -B and -DR, respectively.

In this study, the standard immunosuppressive protocol for all adult kidney transplant recipients consisted of a CNI (cyclosporine A (CsA) or tacrolimus), mycophenolate mofetil (MMF) and corticosteroids. From 2007, induction treatment with an interleukin 2 receptor (IL-2) antagonist (basiliximab) was added to this regimen. Initial CsA C2 target levels were 900 to 1100 μg/L, tapered to 800 μg/L by two months and thereafter CsA C0 levels were 100 to 200 μg/L with gradual reduction towards CsA C0 of 75 to 125 μg/L in the long-term maintenance phase. From January 2007, tacrolimus was introduced as first choice CNI for recipients younger than 50 years of age without known impaired glucose tolerance, and trough levels were targeted at 3 to 7 μg/L [17]. Recipients older than 50 years and recipients with pre-transplant impaired glucose tolerance still received CsA as first choice CNI. Throughout the study, MMF one gram twice daily in CsA recipients and 750 mg twice daily in tacrolimus recipients was used.

Kidneys from deceased donors are accepted at our center without any upper age limit. The decision to accept an organ is made based on an overall judgment of the medical condition of the donor, including hypertension, diabetes, malignancies and age, actual kidney function (diuresis, creatinine and urea concentrations, estimated glomerular filtration rate > 60 mL/min) and results of chemical and microscopic urine analyses. No patient in the study received an organ from a non-heart-beating donor.

A two-sided unpaired *t*-test or Mann–Whitney *U*-test was used, as appropriate, to compare groups. Fisher’s exact test was used to analyze binary data. Uni- and multivariable logistic regression models were used to evaluate variables associated with the development of acute rejection episodes. Survival analyses were performed using the Kaplan-Meier method and Cox proportional hazard models with uncensored and death-censored graft survival as outcomes in the analyses. Available variables with suspected association with the outcome were first tested in univariable models. Variables with possible associations defined as a *P*-value ≤ 0.2 in the univariable model, were then included in the final multivariable models. Recipient age and gender were implemented in all models regardless of the results in the univariable model. There were no missing values in the dataset for any of the variables included in the multivariable Cox models. Accordingly, no patient was excluded from the multivariable models because of missing values. Two patients were excluded in the acute rejection logistic regression model because of missing values for cold ischemia time. All statistical analyses were performed using the statistical software package IBM SPSS Statistics® (version 19.0; IBM, Armonk, NY, USA).

## Results

Between 2004 and the end of 2009, 1,593 kidney transplants were performed at our center. A total of 241 (14%) first-transplant recipients received a kidney from a deceased donor older than 60 years. Seven recipients did not receive CNI at transplantation, one received combined heart and kidney transplants and one received two kidneys. These recipients were excluded, leaving 232 recipients to be included in the final analyses. The majority received CsA (N =206); 164 recipients (71%) were male. The median age of recipients at time of transplantation was 64.6 years (range 14.9 to 82.8 years). Median donor age was 67.0 years (60.2 to 89.0 years). One hundred and forty-nine recipients were treated with IL-2 antagonist (IL-2 plus) versus 83 recipients without IL-2 antagonist treatment (IL-2 minus). Median follow-up was 4.4 years (range 0 to 8.9 years). Recipient age and sex, time on dialysis before transplantation, HLA-mismatches (−A, -B, -DR), cold ischemia time (CIT) and DGF did not differ between the groups. The proportion of cytomegalovirus (CMV)-positive recipients and male donors, as well as the donor age, was significantly higher in the IL-2 plus group. Baseline characteristics are presented in Table 1. At 10 weeks after transplantation, patients in the IL-2 plus group had significantly lower CsA C0 concentrations than those in the IL-2 minus group. Details of immunosuppression at 10 weeks are presented in Table 2. Seventy-nine patients died during the follow-up. Causes of death were cardiovascular diseases in 31 patients, infection in 22 patients, malignancy in 10 patients, gastro-intestinal diseases in 6 patients and miscellaneous in 10 patients. The causes of death were balanced between the groups.

**Table 1:** Transplantation characteristics and immunosuppressive treatment

	**IL-2 plus**	**IL-2 minus**	***P***
	**(N=149)**	**(N=83)**	
Characteristics			
Recipient gender (male)	105 (71%)	59 (71%)	ns
Recipient age (years); median (range)	64.5 (14.9 to 82.8)	65.8 (32.1 to 80.5)	ns
Donor gender (male)	100 (67%)	38 (46%)	0.002
Donor age (years); median (range)	67.6 (60.2 to 89.0)	65.5 (60.3 to 78.8)	0.03
Time on dialysis (years); median (range)	1.5 (0.0 to 6.3)	1.4 (0.0 to 5.9)	ns
Diabetic nephropathy	14 (9%)	8 (10%)	ns
Vascular nephropathy	40 (27%)	32 (38%)	ns
PRA positive recipients	10 (7%)	4 (5%)	ns
Any HLA-A mismatch	122 (82%)	62 (75%)	ns
Any HLA-B mismatch	130 (87%)	78 (94%)	ns
Any HLA-DR mismatch	82 (55%)	41 (49%)	ns
CMV-positive recipient	125 (84%)	57 (69%)	0.01
CMV-positive donor	121 (82%)	72 (87%)	ns
Cold ischemia time (hours); mean ± SD	14.4 ± 5.1	13.3 ± 4.9	ns
Transplant year			
2004	0	24	
2005	6	26	
2006	6	26	
2007	43	6	
2008	46	0	
2009	48	1	
Initial CNI			
CsA	126 (85%)	80 (96%)	0.008
Tacrolimus	23 (15%)	3 (4%)	0.008
PRA, panel-reactive antibodies			

**Table 2:** Follow-up variables; immunosuppression, outcome and cause of graft loss

	**IL-2 plus**	**IL-2 minus**	***P***
	**(N=149)**	**(N=83)**	
Immunosuppression at 10 weeks			
CsA	99 (67%)	60 (77%)	ns
Tacrolimus	31 (21%)	10 (13%)	ns
mTOR inhibitor	14 (10%)	9 (12%)	ns
CsA dose (mg/day)	250.5 ± 73.5	255.4 ± 87.8	ns
CsA C0 concentration	151.4 ± 56.5	183.3 ± 85.6	0.01
Tacrolimus dose (mg/day)	5.65 ± 2.95	8.70 ± 4.64	ns
Tacrolimus concentration	7.3 ± 2.1	9.2 ± 2.3	ns
Prednisolone dose (mg/day)	12,4 ± 5.6	13.6 ± 5.6	ns
MMF (mg/day)	1678.1 ± 402.9	1750.0 ± 619.9	ns
Outcome			
Delayed graft function	53 (36%)	29 (35%)	ns
Acute rejection 90 days post transplant	29 (20%)	34 (41%)	0.001
Acute rejection 180 days post transplant	38 (26%)	35 (42%)	0.01
Steroid-resistant rejection (biopsy proven)	5 (3%)	18 (22%)	< 0.001
C4D positive biopsy	4 (5%)	4 (3%)	ns
Cause of graft loss			
Death with functioning graft	32 (22%)	24 (29%)	ns
Primary non-function	0	6 (7%)	0.002
Primary vascular thrombosis	3 (2%)	2 (2%)	ns
Rejection	5 (3%)	10 (12%)	0.02
Recurrent primary disease	0	1 (1%)	ns
Urological complications	1 (1%)	0	ns
*De novo* glomerulonephritis	1 (1%)	0	ns
Insufficient graft function	0	2 (2%)	ns
Not specified	2 (1%)	2 (2%)	ns

**Figure 1:**
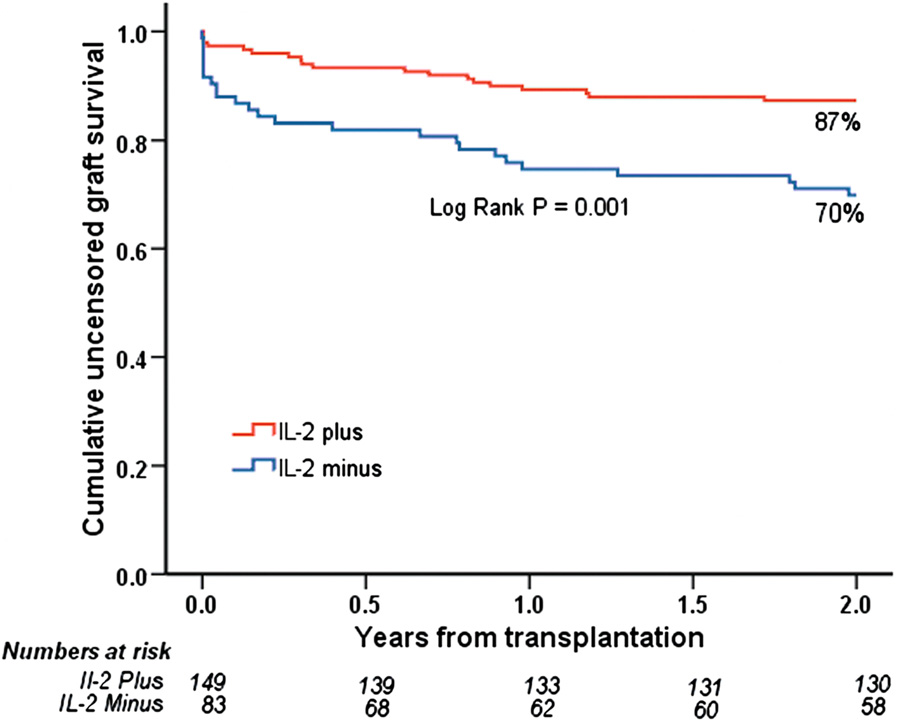
Uncensored graft survival for patients receiving a kidney from a deceased donor older than 60 years, treated with (IL-2 plus, N=149) or without (IL-2 minus, N=83) an IL-2 receptor antagonist.

**Figure 2:**
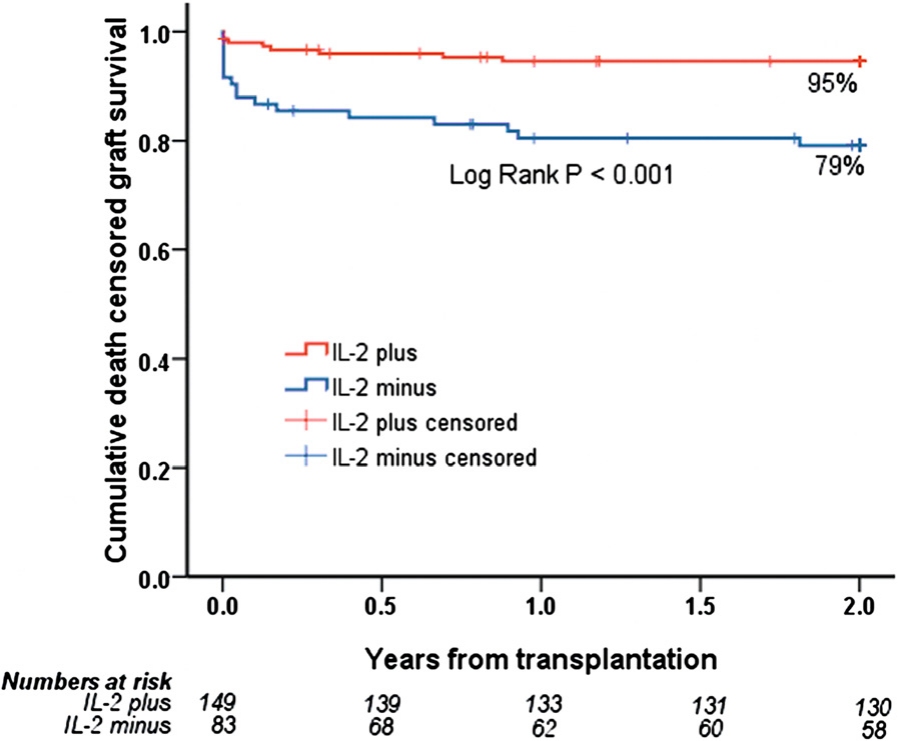
Death-censored graft survival for patients receiving a kidney transplant from a deceased donor older than 60 years, treated with (IL-2 plus, N = 149) or without (IL-2 minus, N = 83) an IL-2 receptor antagonist.

**Figure 3:**
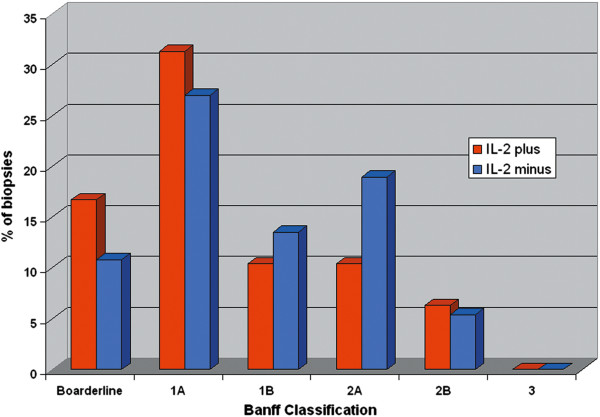
Banff scores of transplant biopsies performed on clinically suspect acute rejection episodes.

In the IL-2 plus group, there were significantly lower rates of acute rejection episodes after 90 days (*P* = 0.001) and 180 days (*P* = 0.01). In addition, the rate of steroid-resistant rejection (*P*< 0.001) was also significantly lower in the IL-2 plus group. We identified a highly significant improvement in two-year uncensored and death-censored graft survival in the IL-2 plus group compared with the IL-2 minus group (Figures 1 and 2). During the observation time, 44 grafts (30%) were lost in the IL-2 plus group versus 47 grafts (57%) in the IL-2 minus group. Rejection data and causes of graft loss are compared in Table 2. There were a total of 136 treated acute rejection episodes. The distribution of transplant indication biopsy Banff scores are presented in Figure 3. Four patients were diagnosed and treated for an acute rejection episode without biopsy-verification. Two patients had non-representative biopsies. Most graft losses occurred during the first 90 days post transplantation, and 90-day uncensored graft survival was 96% and 83% (*P* = 0.001) in the IL-2 plus and IL-2 minus groups, respectively. Among those recipients with a functioning graft at one year after transplantation, serum creatinine values did not differ significantly between the groups; IL-2 plus (N = 121); median 134 μmol/L (64 to313 μmol/L), IL-2 minus (N = 64); median 137 μmol/L (71 to 500 μmol/L). Variables associated with the risk of uncensored and death-censored graft loss two years after transplantation, are presented in Tables 3 and 4. Variables associated with increased risk of an early (first 90 days) acute rejection episode and development of steroid-resistant rejection leading to anti-thymocyte globulin (ATG) treatment are presented in Tables 5 and 6. Steroid-resistant rejection was identified as a possible risk variable for graft loss in the univariable Cox regression models. However, in the multivariable models, steroid-resistant rejection was not significantly associated with the outcome. Because of the association between lack of IL-2 exposure and development of steroid-resistant rejection, increased steroid resistant rejection was considered to be an effect of missing IL-2 exposure and we decided to exclude steroid-resistant rejection from the initial multivariable Cox regression models (Model 1). However, we did perform analyses in a second model (Model 2) and the results of these analyses are shown in Tables 5 and 6. Patients who experienced an acute rejection episode during the first 90 days had significantly impaired kidney function at one year after transplantation compared with those without any rejection episode. Median serum creatinine was 164 μmol/L (range 88 to 500 μmol/L, N = 53) in the rejection group versus 125 μmol/L (64 to 313 μmol/L, N = 132) in the non-rejection group (*P*< 0.001). There was no significant difference in the number of deaths caused by infection between IL-2 minus (12 patients = 14%) and IL-2 plus (7 patients = 5%).

**Table 3:** Two year uncensored graft loss Cox regression analysis

	**Univariable**			**Multivariable model 1**			**Multivariable model 2**		
	**HR**	**95% CI**	***P***	**HR**	**95% CI**	***P***	**HR**	**95% CI**	***P***
Delayed graft function	3.58	1.93 to 6.65	< 0.001	3.12	1.65 to 5.87	< 0.001	3.23	1.82 to 6.09	< 0.001
IL-2 minus	2.63	1.45 to 4.78	0.001	2.28	1.22 to 4.30	0.01	2.02	1.04 to 3.93	0.04
Time on dialysis (per year)	1.28	1.05 to 1.57	0.02	1.27	1.01 to 1.60	0.04	1.27	1.01 to 1.61	ns
Recipient age (per year)	1.03	1.00 to 1.06	0.03	1.02	0.99 to 1.05	ns	1.02	0.99 to 1.05	ns
Any HLA-A mismatch	0.61	0.32 to 1.16	0.1	0.74	0.38 to 1.44	ns	0.68	0.35 to 1.34	ns
Tacrolimus versus CsA	0.34	0.08 to 1.41	0.1	0.44	0.10 to 1.98	ns	0.46	0.10 to 2.11	ns
Recipient gender (male)	1.70	0.82 to 3.54	0.2	1.41	0.66 to 2.99	ns	1.36	0.64 to 2.90	ns
Donor gender (male)	0.72	0.40 to 1.30	0.3	0.80	0.43 to 1.49	ns	0.80	0.43 to 1.50	ns
Steroid-resistant rejection	2.17	1.01 to 4.67	0.05	-	-	-	1.80	0.78 to 4.15	ns

N = 232; 42 graft loss, no missing variables.

The following variables were tested in the univariable model but not included in the multivariable model: CMV-positive donor, CMV-positive recipient, presence of HLA antibodies, donor age, any HLA-B or HLA-DR mismatch, cold ischemia time and presence of acute rejection episode 90 or 180 days after transplantation.

**Table 4:** Two year death-censored Cox regression analysis

	**Univariable**			**Multivariable model 1**			**Multivariable model 2**		
	**HR**	**95% CI**	***P***	**HR**	**95% CI**	***P***	**HR**	**95% CI**	***P***
Delayed graft function	5.21	2.17 to 12.48	< 0.001	5.04	2.05 to 12.42	< 0.001	5.41	2.20 to 13.34	< 0.001
IL-2 minus	4.13	1.78 to 9.57	0.001	4.59	1.91 to 11.02	0.001	3.81	1.53 to 9.49	0.004
Time on dialysis (per year)	1.23	0.94 to 1.63	0.1	1.20	0.89 to 1.62	ns	1.22	0.91 to 1.63	ns
Recipient gender	1.72	0.65 to 4.58	0.3	1.26	0.46 to 3.43	ns	1.23	0.45 to 3.34	ns
Recipient age	1.01	0.98 to 1.05	0.5	1.00	0.97 to 1.03	ns	1.00	0.97 to 1.04	ns
Steroid-resistant rejection	3.65	1.52 to 8.74	0.004				2.52	1.01 to 6.29	0.05

N = 232; 25 graft loss, no missing variables.

The following variables were tested in the univariable model but not included in the multivariable model: CMV-positive donor, CMV-positive recipient, presence of HLA antibodies, donor age, donor gender, any HLA-A, HLA-B or HLA-DR mismatch, type of calcineurin inhibitor (tacrolimus versus CsA), cold ischemia time and presence of acute rejection episodes 90 or 180 days after transplantation.

**Table 5:** Logistic regression analysis: acute rejection episodes during the first 90 days after transplantation

	**Univariable**			**Multivariable**		
	**OR**	**95% CI**	***P***	**OR**	**95% CI**	***P***
IL-2 minus	2.87	1.58 to 5.21	0.001	2.42	1.25 to 4.68	0.009
Any HLA-DR mismatch	1.98	1.09 to 3.60	0.03	2.32	1.19 to 4.52	0.01
Cold ischemia time (per hour)	0.90	0.84 to 0.96	0.001	0.90	0.84 to 0.97	0.003
Tacrolimus versus CsA	0.20	0.05 to 0.86	0.03	0.28	0.04 to 1.01	ns
CMV-positive recipient	0.46	0.24 to 0.90	0.02	0.62	0.30 to 1.30	ns
Time on dialysis (per year)	0.84	0.66 to 1.06	0.14	0.82	0.63 to 1.06	ns
Recipient age	1.00	0.98 to 1.02	0.88	0.98	0.95 to 1.01	ns
Recipient gender (male)	1.46	0.75 to 2.84	0.26	1.46	0.70 to 1.01	ns

N=230; 63 rejection episodes, two patients excluded because of missing variables.

The following variables were tested in the univariable model but not included in the multivariable model: CMV-positive donor, presence of HLA antibodies, donor gender, donor age, delayed graft function, any HLA-A or any HLA-B mismatch.

**Table 6:** Logistic regression analysis: ATG treated steroid-resistant cellular rejection

	**Univariable**			**Multivariable**		
	**OR**	**95% CI**	***P***	**OR**	**95% CI**	***P***
IL-2 minus	7.98	2.84 to 22.41	< 0.001	8.04	2.77 to 23.25	< 0.001
Any HLA-DR mismatch	2.18	0.86 to 5.52	0.1	2.45	0.92 to 6.57	ns
CMV-positive recipient	0.38	0.15 to 0.94	0.04	0.59	0.22 to 1.58	ns
Recipient age	0.99	0.96 to 1.03	0.59	0.99	0.95 to 1.02	ns
Recipient gender (male)	1.55	0.55 to 4.37	0.40	1.29	0.42 to 3.95	ns

N=232; 23 rejection episodes, no missing variables.

Variables tested in the univariable model but not included in the multivariable model: CMV- positive donor, presence of HLA antibodies, donor gender, donor age, type of calcineurin inhibitor (tacrolimus versus CsA), any HLA-A or HLA-B mismatch, time on dialysis, cold ischemia time and delayed graft function.

## Discussion

In this single center retrospective analysis of recipients of organs from deceased donors older than 60 years, the use of IL-2 antagonist induction was associated with improved short and intermediate (two-year) graft survival. Outcome was not associated with recipient age or HLA-matching. We identified a lower early acute rejection rate and a lower rate of steroid-resistant rejection episodes when IL-2 antagonists were used. It is noteworthy that the donor age was higher in the IL-2 plus group than in the IL-2 minus group. This may indicate that the negative effect of increased donor age can be reduced by more aggressive immunosuppressive therapy, such as induction treatment with an IL-2 antagonist. Greater donor age and time on dialysis prior to transplantation have previously been shown to negatively affect graft survival in older recipients [18]. The number of grafts with primary non-function differed between the groups (Table 2). This cannot be explained by introduction of any new preservation methods or surgical techniques. When the patients with primary non-function were removed from the models, the results were robust in all models, except in the uncensored graft loss model.

The use of ECD to increase the available donor pool has been reported to result in impaired but acceptable outcomes when compared with outcomes using standard criteria donors [19-21]. Studies describing the use of IL-2 antagonists in transplantation with ECD are scarce. Webster *et al*. for the Cochrane Collaboration [7] identified 32 studies with a total of 5,854 adult recipients where IL-2 antagonists were compared with placebo. However, none of these studies focused on IL-2 antagonists and the use of ECD kidneys or kidneys from older deceased donors. They found that graft loss, including death with a functioning graft, was reduced by 25% at six months and one year but not beyond that. As seen in our study, the incidence of early biopsy-proven acute rejection episodes was also reduced. Webster *et al.* also compared IL-2 antagonist induction with ATG induction [7] and found no difference between these two regimens for graft loss at any time or for clinically diagnosed acute rejection. However, they described a 75% increase in malignancy and a 32% increase in CMV disease among patients who received ATG compared with IL-2 antagonist induction. Others have described reduced patient survival after ATG induction in older (> 60 years) recipients given doses > 6 mg/kg [22].

At our center, we have historically experienced a relatively high rate of acute cellular rejection episodes [5]. Recipients treated with IL-2 receptor antagonist induction had a significantly lower risk of experiencing an acute cellular rejection. Perhaps even more important in an elderly population is that this induction treatment was strongly and significantly associated with a reduced rate of steroid-resistant rejection. Treatment of steroid-resistant rejection episodes with ATG may be associated with several serious complications [7,23], especially in a group of older patients who are receiving the majority of their organs from older donors [22]. We therefore regard this *protective effect* as very important because it leads to fewer rejection episodes and, in particular, fewer steroid-resistant rejection episodes.

As a result of the logistic regression analysis presented in Table 6, we considered that the apparent effect observed for steroid-resistant rejection was in fact mediated by lack of IL-2 exposure and was therefore a part of the causal pathway of graft loss. Accordingly, steroid-resistant rejection did not satisfy the criteria for a potential confounder [24] and was omitted from the initial multivariable models. This consideration was supported by the result of new analyses in which these two variables were tested separately and thereafter together in the same model. In these analyses we observed that the effect estimates for IL-2 receptor antagonist were only slightly changed from the univariable analysis when steroid-resistant rejection was added to the model, whereas the change was considerable for steroid-resistant rejection (Table 7).

**Table 7:** Uni- and multivariable models for testing the interaction effect of IL-2 minus and steroid-resistant rejection

	**Univariable**			**Multivariable**		
	**HR**	**95% CI**	***P***	**HR**	**95% CI**	***P***
Uncensored graft loss						
IL-2 minus	2.68	1.46 to 4.91	0.001	2.46	1.30 to 4.67	0.006
Steroid-resistant rejection	2.23	1.03 to 4.80	0.04	1.46	0.66 to 3.29	0.36
Death-censored graft loss						
IL-2 minus	4.05	1.75 to 9.39	0.001	3.34	1.37 to 8.13	0.008
Steroid-resistant rejection	3.64	1.52 to 8.72	0.004	2.17	0.86 to 5.46	0.10

Because of age-matching policies, most patients receiving kidneys from older deceased donors are themselves older. Concerns have been raised regarding the increased risk of serious infectious complications related to increased immunosuppressive load [13,25]. In the present study, we were not able to detect any increased incidence of fatal infections in the IL-2 plus group. We therefore regard the use of IL-2 antagonist induction as safe, even in older recipients. Unfortunately, we have no data describing the incidence of non-fatal infectious complications during the post transplant period and are therefore not able to compare the incidence of less serious infections.

Compared with those at other transplant centers, the recipients in our study had a relatively short time on dialysis prior to transplantation and the mean CIT was generally short. These are both factors that might be part of the explanation for our outcome. The presence of DGF was previously described as a risk variable for graft loss [10,18]. In this study, we identified DGF as an independent risk variable in both multivariable regression models of graft loss, but we found no difference in the frequency of DGF between the groups (36% versus 35%; Table 1). In the context of marginal donors, any intervention that could preserve the function of the graft and reduce the incidence of DGF would be of importance for the outcome. Improved methods for recovery of organs and graft preservation may therefore further improve the outcome of kidney transplantation with ECD [9].

This study has several limitations. First, it is a retrospective and observational study. However, this design does allow us to generate and test a hypothesis by identifying significant associations between several variables and the outcomes. The potential to make causal inferences is as a rule considerably less than in a randomized controlled trial [26,27]. Our database is, however, complete with respect to all the variables and events included in the models. Second, the size of our cohort is relatively small and the patients were allocated to the two treatment groups based on the standard protocol at the time of transplantation, not as a result of randomization. This introduces another bias, namely the possibility that improvements in medical or surgical treatment might be responsible for the results, not the introduction of an IL-2 antagonist. However, apart from the IL-2 antagonist and the introduction of tacrolimus as the standard CNI for recipients younger than 50 years (Table 1), the standard immunosuppressive protocol has remained virtually unchanged for the whole study period. After the results of the SYMPHONY study in 2007 [17], however, there is reason to believe that some reduction in CNI dose has been introduced, even if the protocol is unchanged. The CNI doses and trough levels presented in Table 2 support this belief. The number of patients receiving tacrolimus (11%) is far too small to make any conclusions about which CNI should be preferred. Neither the surgical procedures nor the procedures for graft recovery and preservation at our center have changed significantly during the period of the study. Accordingly, we consider that the bias related to improved medical or surgical treatment is minor. As shown in Table 1, except for the higher median donor age, the higher rate of male donors and the higher rate of CMV-positive recipients in the IL-2 plus group, the groups were comparable.

This study is important as it addresses the utilization of kidneys from older donors. Most studies performed addressing modern immunosuppression regimens include younger recipients and do not focus on donor age. There is a lack of representation of older patients in randomized controlled trials [28]. The US Food and Drug Administration continues to encourage us to study the effects of new and old immunosuppressive regimens in older patients [29]. In organ transplantation, there is also a need to focus on donor age.

In this study, we only investigated recipients of kidneys from deceased donors older than 60 years of age. ECD kidneys from deceased donors 50 to 59 years of age with defined medical criteria [6] were not included in the analysis. Our results are therefore representative for kidneys transplanted from older deceased donors and not automatically applicable to all ECDs. We do however believe that these older donors are the most important reserve of organs available for kidney transplantation today, and so have the largest potential for expanding the donor pool.

Finally, the relatively short observational period, which excludes us from making any conclusions about the long-term prognosis, is also an important limitation. However, previous studies have shown an obvious linear relationship between short- and long-term graft survival [12,19,30].

## Conclusions

Induction treatment with IL-2 antagonist is associated with a reduced incidence of acute rejection episodes and improved two-year graft survival in patients receiving a kidney from a deceased donor older than 60 years. Optimizing immunosuppressive regimens is important, and adding an IL-2 antagonist to treatment of recipients of kidneys from older deceased donors may be a way to improve the outcomes. As outcomes improve, increased utilization of kidneys from older deceased donors may be an important contribution to alleviation of the scarcity of donor organs for kidney transplantation.

## Abbreviations

ATG: Anti-thymocyte globulin; CI: Confidence interval; CIT: Cold ischemia time; CMV: Cytomegalovirus; CNI: Calcineurin inhibitor; CsA: Cyclosporine A; DGF: Delayed graft function; ECD: Expanded criteria donor; ESRD: End stage renal disease; HLA: Human leukocyte antigen; HR: Hazard ratio; IL-2: Interleukin 2; MMF: Mycophenolate mofetil; mTOR: Mammalian Target of Rapamycine; OR: Odds ratio; PRA: Panel reactive antibodies

## Competing interests

The authors declare that they have no competing interests.

## Authors’ contributions

KH was responsible for design of the study, statistical analyses and is the main author of the manuscript. ST participated in the statistical analyses, evaluation of results, manuscript revision and approved the final manuscript. AH contributed to the study design, evaluation of results, and manuscript revision and approved the final manuscript. TL contributed to the study design and was responsible for collection of data from the Norwegian Renal Registry. He also contributed to manuscript revision and approved the final manuscript. AVR contributed to the study design, evaluation of results, and manuscript revision and approved the final manuscript. AF contributed to the study design, evaluation of results, and manuscript revision and approved the final manuscript. KM was the main supervisor of the study and contributed to the study design, collection of data, evaluation of results, and manuscript revision and approved the final manuscript. All authors read and approved the final manuscript.
